# A Network Pharmacology Approach to Explore the Potential Mechanisms of Yifei Sanjie Formula in Treating Pulmonary Fibrosis

**DOI:** 10.1155/2020/8887017

**Published:** 2020-11-30

**Authors:** Bo Qiao, Yueying Wu, Xiaoya Li, Zhenyuan Xu, Weigang Duan, Yanan Hu, Wenqing Jia, Qiuyang Fan, Haijing Xing

**Affiliations:** ^1^School of Basic Medical Science, Yunnan University of Chinese Medicine, Kunming, Yunnan, China; ^2^College of First Clinical Medical Science, Yunnan University of Chinese Medicine, Kunming, Yunnan, China; ^3^School of Chinese Medical Science, Hunan University of Chinese Medicine, Changsha, Hunan, China; ^4^College of Basic Medical Sciences, Guangzhou University of Chinese Medicine, Guangzhou, Guangdong, China; ^5^Provincial Innovation Team of Yunnan University of Chinese Medicine for Traditional Chinese Medicine to Regulate Human Microecology, Kunming, Yunnan, China

## Abstract

**Objective:**

Yifei Sanjie Formula (YFSJF) is an effective formula on pulmonary fibrosis (PF), which has been used in clinic for more than 30 years. In order to investigate the molecular mechanism of YFSJF in treating PF, network pharmacology was used to predict the cooperative ingredients and associated pathways.

**Methods:**

Firstly, we collected potential active ingredients of YFSJF by TCMSP databases. Secondly, we obtained PF-associated targets through OMIM and Genecards database. Finally, metascape was applied for the analysis of GO terms and KEGG pathways.

**Results:**

We screened out 76 potential active ingredients and 98 associated proteins. A total of 5715 items were obtained by GO enrichment analysis (*P* < 0.05), including 4632 biological processes, 444 cellular components, and 639 molecular functions. A total of 143 related KEGG pathways were enriched (*P* < 0.05), including IL-17 signaling pathway, T cell receptor signaling pathway, TNF signaling pathway, calcium signaling pathway, TH17 cell differentiation, HIF-1 signaling pathway, and PI3K-Akt signaling pathway.

**Conclusion:**

YFSJF can interfere with immune and inflammatory response through multiple targets and pathways, which has a certain role in the treatment of PF. This study lays a foundation for future experimental research.

## 1. Introduction

Pulmonary fibrosis (PF) is a common and irreversible pathological outcome of many chronic lung diseases, which is characterized by progressive alveolar epithelial injury and abnormal repair. In recent years, the incidence rate and mortality rate of PF are increasing [1]. It is reported that the annual incidence rate of the disease is 4.6∼16.3 per 100,000 and the prevalence rate is 13∼20 per 100,000 [2]. At present, the pathogenesis of PF is not completely clear. The main clinical treatment can be made with glucocorticoids, immunosuppressants, antioxidants, and biological agents [3]. However, these drugs cannot effectively reverse the development of PF and bring multiple side effects to some extent. Therefore, it is necessary to explore other treatments in order to both control the symptoms of the patients and extend their life span.

Yifei Sanjie Formula (YFSJF) is an effective formula in treating PF, which is authorized by professor Qingshen Li, a famous physician of traditional Chinese medicine in Yunnan Province. The formula is based on Yupingfeng formula, which is composed of Sangbaipi (*Mori Cortex*), Zhebeimu (*Fritillariae Thunbrgii Bulbus*), Jiezi (*Sinapis Semen*), E'zhu (*Curcumae Rhizoma*), and Sanqi (*Panax Notoginseng (Burk.) F. H. Chen Ex C. Chow*). In the whole formula, drugs are compatible with each other to achieve the effects of tonifying qi, eliminating phlegm and removing blood stasis. The preliminary research of the research group confirmed [4, 5] that the formula can promote the metabolism and absorption of extracellular matrix such as collagen fibers and then alleviate the development of PF. Modern pharmacological studies have found that most of Chinese medicine in YFSJF can alleviate PF by improving autoimmune function [6], relieving excessive inflammation, inhibiting apoptosis, and reducing collagen deposition [7]. However, the specific mechanism needs to be further studied.

**Figure 1:**
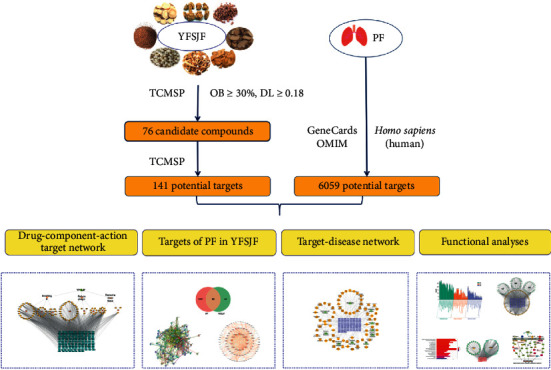
Flow chart of network pharmacology study on the mechanism of action of YFSJF in the treatment of PF.

Network pharmacology is based on high-throughput group data analysis, computer virtual computing, and network database retrieval [8]. The relationships between components, proteins, and related pathways are expounded on the whole as well as explained their material basis and functions mechanism [9]. Therefore, the application of network pharmacology to the study of the mechanism of action of TCM has unique advantages and great potential for development. In this study, network pharmacology was used to obtain the main ingredients, potential targets and pathways of YFSJF in the treatment of PF. The detailed workflow is shown in Figure 1.

## 2. Materials and Methods

### 2.1. Collection of Potential Active Ingredients and Associated Targets

The active ingredients and corresponding targets of YFSJF were collected from Traditional Chinese Medicine Systems Pharmacology database [10] (TCMSP database, https://tcmspw.com/tcmsp.php) under the conditions of oral bioavailability (OB) ≥30% and drug-like (DL) ≥0.18. The targets data in the TCMSP database were collected from the DrugBank database [11] (https://www.drugbank.ca/). The UniProt database [12] (https://www.uniprot.org/) was utilized to convert the obtained target names of YFSJF to the gene names.

### 2.2. Construction of the YFSJF-Active Ingredient-Action Target Network

The active ingredients in YFSJF and their corresponding gene names were import into Cytoscape software [13] (version3.7.1, http://www.biostars.org/p/260759) to construct the YFSJF-active ingredient-action target network and were analyzed with the tool Network Analyzer.

### 2.3. Prediction of Potential Targets of PF Treated by YFSJF

The GeneCards database [14] (https://www.genecards.org/) and the Online Mendelian Inheritin man database [15] (OMIM, https://omim.org/) provide functional information for all known and putative human genes. Using the “PF” as the keyword, the species was designated as “*Homo sapiens* (Human)” to obtain the associated targets of the disease.

The targets associated with the ingredients of YFSJF and those associated with PF were analyzed with the Venn diagram package in R Program, and a Venn diagram was made. The intersection of the targets both from the two groups was served as the potential targets of YFSJF on PF.

### 2.4. Construction Target-Disease Network

In order to get a better understanding of diseases related to YFSJF, the potential targets were projected to the Therapeutic Target Database [16] (TTD, http://db.idrblab.net/ttd/) to collect their corresponding diseases. Then, diseases were classified into groups according to the Medical Subject Headings Browser [17] (MESH Browser, https://www.ncbi.nlm.nih.gov/mesh/?term=). Finally, target-disease network was constructed based on potential targets and their corresponding diseases. The importance of nodes with “Degree” was constructed with Network Analyzer.

### 2.5. Construction YFSJF-PF PPI Core Network

The obtained potential targets were imported to the STRING database [18] (https://string-db.org/), the protein species was set as “HOMA sapiens”, and the minimum interaction threshold was set as “medium confidence” (>0.4). Next, Network Analyzer was used to evaluate the importance of the nodes with “Degree”. The final network was constructed based on the nodes with positive degree, and then, the network was exported to an image.

### 2.6. Enrichment of Gene Ontology (GO) and Kyoto Encyclopedia of Genes and Genomes (KEGG) Pathway

The potential targets of YFSJF on PF were introduced into the Metascape [19] (https://metascape.org/gp/index.html#/main/step1) for enrichment analysis. *P* < 0.05 was accepted to obtain main GO and KEGG pathways, and the first 20 pathways were screened out. Afterwards, Cytoscape software was used to analyze the enrichment of GO and KEGG pathways for potential targets, and the relationship between potential targets and biological functions or disease-related pathways was screened out and visualized.

## 3. Results

### 3.1. Information of Potential Active Ingredients and Their Targets in YFSJF

**Figure 2:**
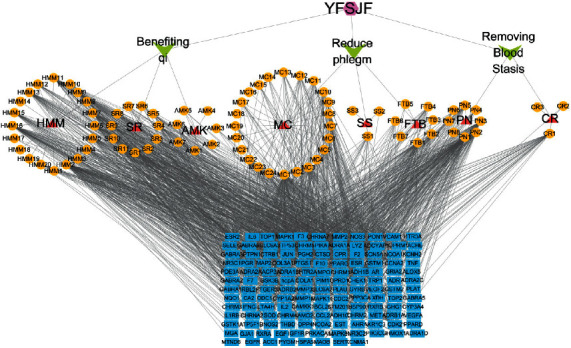
The drug-component-action target network. Composited of 1 Formula (purple), 3 efficiency (green), 8 Chinese medicinal materials (red), 76 compounds (yellow), and 141 targets (blue).

A total of 76 active ingredients of YFSJF with OB ≥30% and DL ≥0.18 were obtained from the TCMSP database. Among them 20 ingredients were from *Hedysarum Multijugum Maxim (HMM)*, 11 from *Saposhnikoviae Radix (SR),* 7 from *Atractylodes Macrocephala Koidz (AMK)*, 7 from *Fritillariae Thunbrgii Bulbus (FTB)*, 3 from *Sinapis Semen (SS)*, 24 from *Mori Cortex (MC)*, 8 from *Panax Notoginseng (Burk.) F. H. Chen Ex C. Chow (PN)*, and 3 from *Curcumae Rhizoma (CR)* (Supplementary Table 1). All active ingredients were entered into the TCMSP database, and a total of 141 targets were found. Then, a visual YFSJF-active ingredients target network was constructed by using Cytoscape3.7.1 (Figure 2), containing 236 nodes and 1552 edges, and showed that the mean degree of candidate ingredients was 16.01. Among them, 31 ingredients were with degree value higher than 16.01. The top 10 active ingredients in degree value are as shown in Table 1. Especially, quercetin, kaempferol, and beta-sitosterol act on 88, 56 and 52 targets, respectively, which were located in the center of the drug-component-action target network and therefore are representative active ingredients of YFSJF. As for the targets, ESR, AR, PTGS2, F2, and PPPARG were targeted by 62, 57, 51, 42, and 42 compounds of YFSJF, respectively, which indicated that these targets might be largely involved in the underlying action mechanisms of YFSJF.

### 3.2. Protein Interaction Network (PPI) and Core Network Analysis

**Figure 3:**
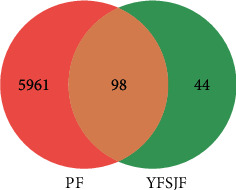
Venn diagram of targets of PF treated by YFSJF. A total of 98 overlapped genes between the YFSJF and PF were obtained.

**Figure 4:**
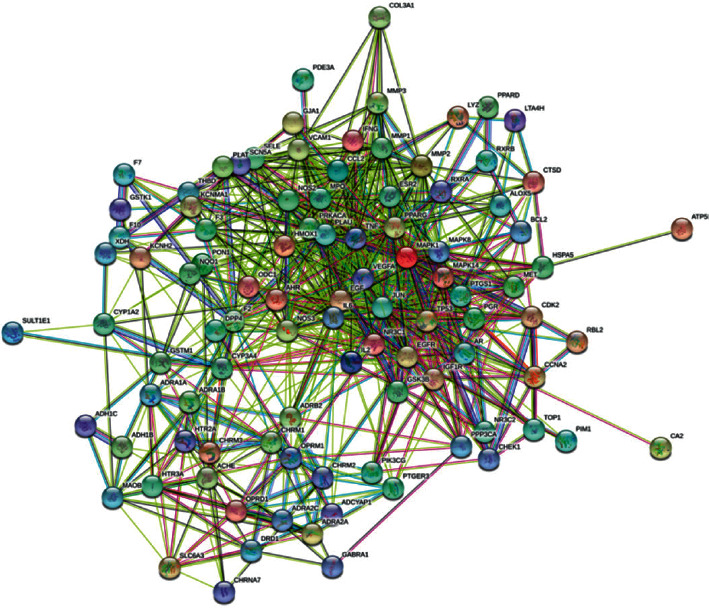
The protein-protein interaction (PPI) network.

**Figure 5:**
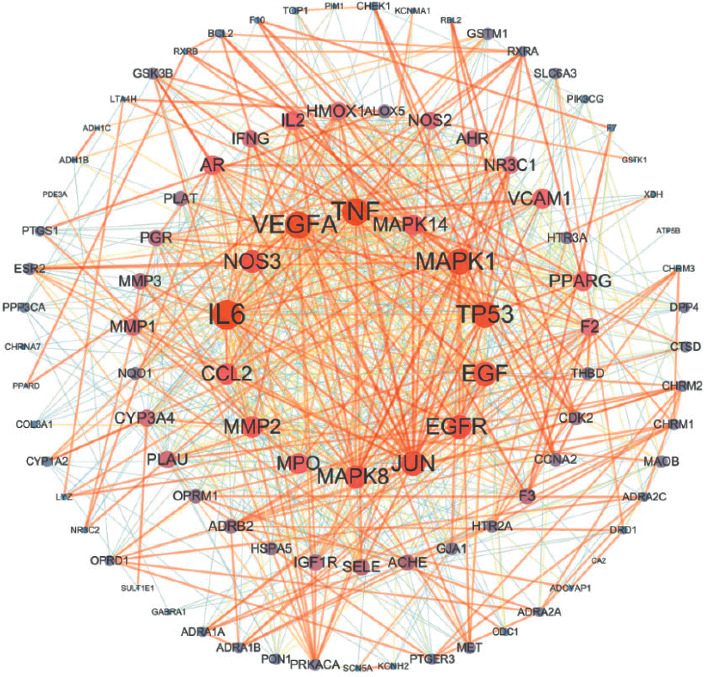
The bar plot of the protein-protein interaction (PPI) network. The node size and color represent the importance of the node in the network.

Through GeneCards and OMIM database, a total of 6059 PF disease targets were obtained. Through the Venn diagram package in R Program, the 6059 disease targets and the 141 active ingredient targets were used to draw a Venn diagram. And 98 overlpped targets were obtained for both (Figure 3). In order to study the interaction between the potential targets in vivo and find the key targets, the 98 overlapped targets were analyzed based on the String database. The genes corresponding to the target proteins were input to the String database, and a total of 98 nodes and 806 edges with an average degree of 16.4 (Figure 4) were obtained. Through the Network analyzer tool, we analyzed the network that the node was bigger and the color was redder and the degree of the protein was larger (Figure 5). According to the Figure 5, IL6, TNF, VEGFA, TP53, MAPK1, JUN, and EGF are the main targets of the active ingredients of YFSJF. It was suggested that the mechanism of YFSJF for treating PF was closely related to these core targets. In summary, YFSJF played a cooperative role in treating PF through multiple potential targets.

### 3.3. Target-Disease Network of YFSJF

**Figure 6:**
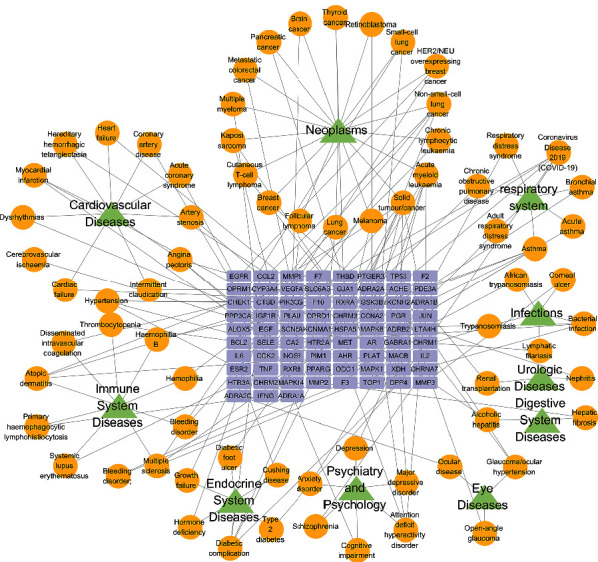
Target-disease network. A total of 65 target genes (blue) were connected to 71 diseases (yellow), which were divided into 14 categories (green) according to Medical Subject Headings.

Target-disease (T-D) network was constructed based on the potential targets and corresponding diseases. As shown in Figure 6, T-D network included 156 nodes (75 targets, 71 target-related diseases, and 10 disease categories) and 181 target-disease interactions. The 71 diseases can be classified into 10 groups according to the MeSH Browser. Most of the collected diseases belonged to neoplasms (18/71), followed by cardiovascular diseases (12/71), immune system diseases (10/183), and respiratory diseases (6/71), which suggested that YFSJF might act on multiple diseases. Moreover, PF is the common pathological outcome of many chronic lung diseases, and the results showed that the potential targets were related to respiratory diseases including asthma, acute asthma, bronchus asthma, coronavirus disease 2019 (covid-19), respiratory disease syndrome, chronic obstructive PF, and adult respiratory disease syndrome. The above results supplied a systematic evidence for the TCM theory that YFSJF had extensive pharmacological activities and was successfully applied to the treatment of various diseases.

### 3.4. GO Enrichment Analysis

**Figure 7:**
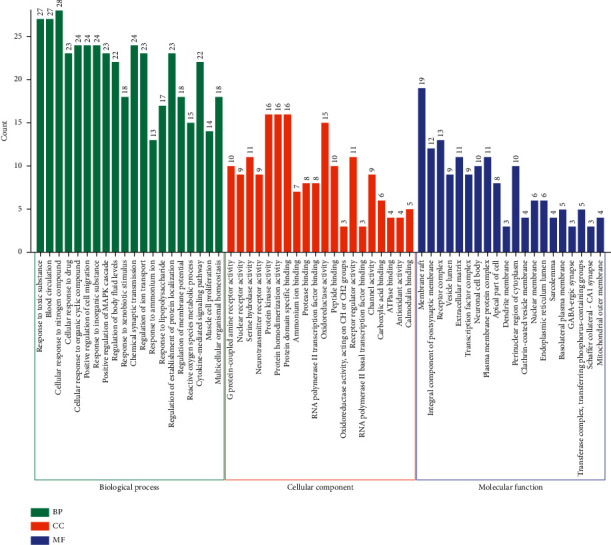
GO analysis of candidate targets of YFSJF against PF. The top 20 GO functional categories with *P* < 0.05 were selected. The *X*-axis represents the significant enrichment counts of these terms, while the *Y*-axis represents the categories of “biological process” in the GO of the target gene.

**Figure 8:**
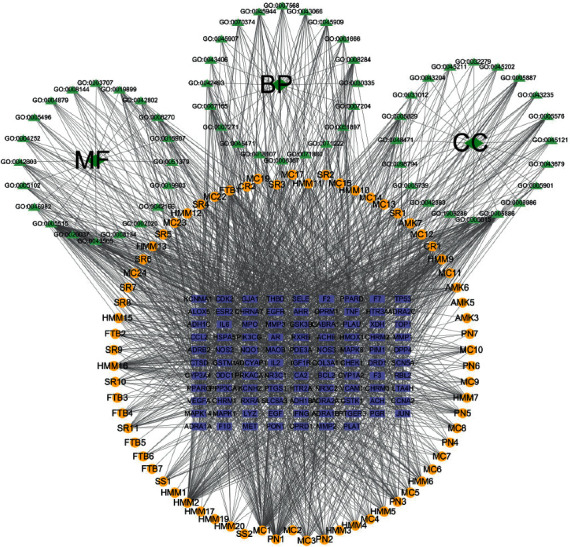
GO-GENE-components network. In the figure, blue rectangle represents genes, yellow cycles represent ingredients, and green triangles represent BP, CC, and MF.

GO function of 98 action targets (*P* < 0.05) was enriched with Metasape. Top 20 terms are shown in Figure 7 (Supplementary Table 2). The results showed that the main biological processes were involved in cellular response to nitrogen compound, response to toxic substance, blood circulation, cellular response to organic cyclic compound, and positive regulation of cell migration, and response to inorganic substance; the cell locations were membrane raft, receptor complex, integral component of postsynaptic membrane, extracellular matrix, and plasma membrane protein complex. The molecular functions they play were protein kinase activity, protein homodimerization activity, protein domain-specific binding, oxidoreductase activity, serine hydrolase activity, and receptor regulator activity. Then, the GO-gene network was constructed (Figure 8). It was manifested that the targets of YFSJF were mainly involved in the regulation of cell growth and differentiation, repair of tissue damage, regulation of innate immunity and adaptive immunity, and initiation of apoptosis (killing tumor cells, virus infected cells, or activated T cells). The effects on PF could be achieved through the regulation of cell differentiation and gene expression and regulation of immune system and autonomic nervous system.

### 3.5. Pathway Enrichment Analysis

**Figure 9:**
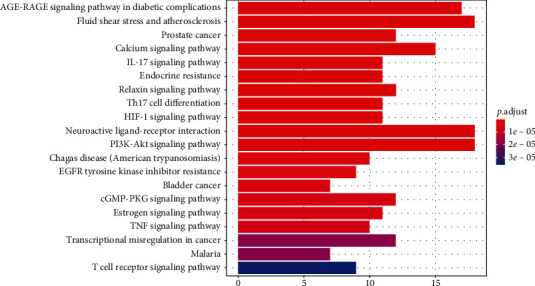
Kyoto Encyclopedia of Genes and Genomes (KEGG) pathway enrichment candidate targets of YFSJF against PF. The top 20 pathway categories with significant (*P* < 0.05) were selected. The *X*-axis represents the significant enrichment counts of these terms, and the *Y*-axis represents the main pathway.

**Figure 10:**
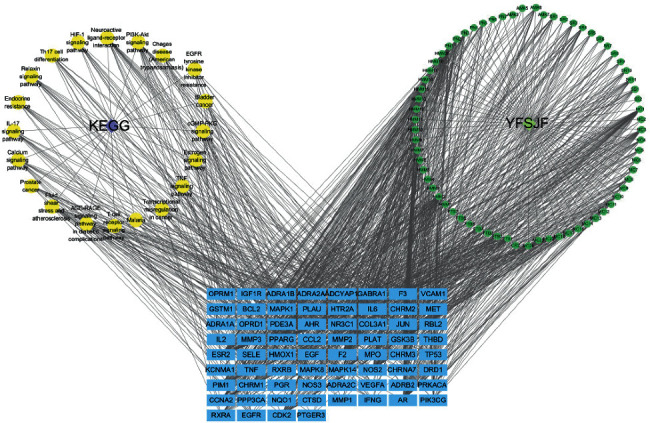
Pathway-gene network. In the figure, rectangle represents genes (red), circular represent pathway (green), and circular represent components (blue).

The Metasape was used to perform KEGG pathway enrichment analysis based on the 98 targets. According to the results with significance (*P* < 0.05), enrichment analysis yielded 144 pathways and visualization analysis of the top 20 signaling pathways (Figure 9), including calcium signaling pathway, IL-17 signaling pathway, TNF signaling pathway, PI3K-Akt signaling pathway, and Th17 cell differentiation. In addition, the pathway-gene network was constructed to further prove the core gene representatives that played an important role in the pathways (Figure 10). As shown, YFSJF can affect multiple pathways through multiple targets, playing a role in regulating immunity, alleviating inflammation, and then intervening in the occurrence and development of PF at the overall level. The top 20 pathways with their representative enriched terms are specified in Table 2.

## 4. Discussion

TCM is guided by the overall concept and based on the principle of differentiation of symptoms and treatments. TCM plays a synergistic role in the treatment of diseases, removing pathogenic factors, regulating viscera, and supporting vital energy. As a clinical effective formula for treating PF, YFSJF has the effects of tonifying qi, eliminating phlegm and eliminating blood stasis in TCM theory. Despite the remarkable clinical effect of this prescription, no systematic mechanism study has been conducted. Based on the research idea of disease-gene-target-drug, this paper systematically elaborated the mechanism of action of YFSJF from the microscopic molecular point of view by applying the technology of network pharmacology and provided a reference for further research on the mechanism of action of YFSJF against PF.

### 4.1. Therapeutic Effect of Main Active Components of YFSJF on PF

Previous research findings [20], YFSJF has a certain therapeutic effect on PF, which can significantly improve lung tissue damage and reduce the degree of pulmonary fibrosis. The main active quercetin and beta-sitosterol of *Astragalus membranaceus*, *Panax notoginseng*, and *Morus Alba* have antioxidant and anti-inflammatory effects. Studies [21, 22] have shown that quercetin can inhibit AKT/mTOR signaling pathway, reduce inflammatory response and collagen deposition, and alleviate PF by downregulating IL-6, IL-8, TGF-beta, NF-kB, COL-1, COL-3, and VEGF as well as ATG5 and CL3. The ingredient can also inhibit the expression of TGF-beta 1 and miR-21, enhance the activity of Smad7, and inhibit the TGF-beta 1/Smads signaling pathway, thereby alleviating the inflammatory response and collagen deposition. Beta-sitosterol can significantly reduce LPS-induced pulmonary edema and inflammatory response and reduce the release of TNF-a and IL-6 [23]. YU et al [24] found that beta-sitosterol could alleviate PF by inhibiting EMT through inhibiting the TGF-beta 1/Snail pathway to alleviate PF. Yang et al [25] found that Ivy saponin can reduce inflammatory injury of endothelial cells and protect vascular endothelial cells from inflammation injury by regulating the NF-kappa B signaling pathway, inhibiting the adsorption of inflammatory factors and adhesion molecules. Based on the study, the ingredients of YFSJF can act on multiple targets and play a synergistic role, which may be the material basis of YFSJF in the treatment of PF.

### 4.2. Molecular Mechanism Analysis of YFSJF Based on Pathogenesis of PF

According to the research report on the active ingredients of YFSJF, it was indicated that the prevention and treatment of PF mainly involved PI3K/AKT signaling pathway, HIF-1 signaling pathway, TNF signaling pathway, IL-17 signaling pathway, T cell receptor signaling pathway, calcium signaling pathway, and Th17 cell differentiation. It played roles in regulation on immune, inhibition on inflammation, and alleviation on the occurrence and development of pulmonary fibrosis.

Long-term or repetitive cellular damage in pulmonary fibrosis initiates immune-related damage repair process, resulting in the proliferation of abnormally activated fibroblasts [26]. KEGG enrichment analysis showed that IL-17 signaling pathway, T cell receptor signaling pathway, calcium signaling pathway, Th17 cell differentiation pathway, and other pathways were obtained. Many literatures found that the above pathways were related to immune response, indicating that YFSJF could play an immune regulatory role through multiple pathways.

T cells are important immune cells, which mediate adaptive immune responses in humans and express calcium ions in T lymphocyte release activates Ca^2+^ release-activated Ca^2+^ channel, transient receptor potential channel, purinergic receptor), and voltage-gated potassium channel subfamily A member 3 and other ion channels [27]. The activity of these ion channels can regulate the electrophysiological activity of immune cells and then effectively regulate the activity of immune cells. Calcium ion channels directly participate in the regulation of intracellular signal transduction process by regulating the change of intracellular calcium concentration and then regulate the immune response [28]. Calcium ions, as second messengers, play an important role in the immune response of immune cells. They participate in the regulation of intracellular signal transduction processes. Therefore, regulating the concentration of calcium ions in cells is important to maintain the normal function of immune cells. Studies have shown that knocking out ORAI1 in mouse T cells or human T cells weakens calcium influx in cells and severely impedes adaptive immune responses in mice or humans. Th17 cells, a distinct lineage of activated CD4^+^ T cells, are raised in the airways and lungs of PF patients, upregulating tissue inflammation, and exacerbate alveolar destruction by producing IL-17, an important pathogenic factor. Th17 cells also exert direct influence on epithelial cells, smooth muscle cells, and airway fibroblasts to induce neutrophil chemokine secretion [29, 30]. Quercetin-3-*β*-D-glucopyranosyl-7-O*β*-D-gentiobioside (QGG) can significantly improve the survival conditions, growth ratio, and pulmonary functions of rats, which can reduce the ratio of CD4^+^ IL-17^+^/FOXP3^+^CD4^+^ in peripheral blood and reduce the apoptosis of lung tissues, repair damaged tissue, and maintain the integrity of organ [31]. Th17 cells can produce a large amount of IL-17, and the most characteristic members of the IL-17 family, IL-17A, and IL-17F, form homodimers or heterodimers to bind the heterodimer IL-17RA and IL-17RC complexes and activate downstream signaling to achieve biological effects such as host defense or the pathogenesis of autoimmune diseases and other inflammatory diseases [32]. Baldeviano G C et al [33] have found that blocking endogenous IL-17 improves the degree of bleomycin-induced fibrosis in mice, which may be related to the Bax/Bcl-2-mediated mitochondrial apoptosis pathway. Pulmonary inflammation occurs in mice at all times when exposed to cigarette smoke, while IL-17 secreting cells are recruited to promote IL-17 secretion [34]. Anti-IL-17 antibody can reduce the number of neutrophils in mice and the concentration of MUC5AC in bronchoalveolar lavage fluid and attenuate neutrophilic airway inflammation [35]. The studies have shown that quercetin inhibits IL-17-stimulated RANKL production in RA-FLS and IL-17-stimulated osteoclast formation. Quercetin dihydrate induced an insignificant change in the level of IL-2 and IL-6 and slightly increased in IFN-*γ* content [36]. Therefore, YFSJF played an important role in regulating immunity through IL-17 signaling pathway, T cell receptor signaling pathway, calcium signaling pathway, and Th17 cell differentiation.

PF is characterized by alveolar interstitial inflammatory cell infiltration, fibroblast proliferation, and alveolar interstitial fibrous connective tissue deposition. The aggregation of inflammatory cells and the release of cytokines will induce a large number of collagen fibers, resulting in the remodeling of lung tissue structure, reduction of alveolar number, deformation, atresia, and loss of lung function [37]. Therefore, inflammatory response also plays an important role in PF. It has been reported that THF, PI3K/Akt, and HIF-1 pathways are closely related to inflammatory response and interfere with the occurrence and development of PF, which is consistent with the results of this study.

Tumor necrosis factor (TNF) is a kind of cytokines, which can promote cell growth, differentiation, and apoptosis and induce inflammation by binding to specific receptors on the cell membrane [38]. TNF-alpha can induce the release of cytokines such as TGF-beta, IL-1, IL-6, and NF-kappa B to form a cytokine network, which together leads to the occurrence of PF. It was found that nintedanib and BLM-MSCs exerted their anti-inflammatory effect through minimizing the expression of TNF-*α* and IL-6 and decrease in the alveolar wall thickening, both in the inflammatory infiltrate and in the collagen fiber deposition [39]. Hypoxia-inducible factor 1 (HIF-1) widely exists in the human body under hypoxic environment, regulates the body's response to hypoxia, regulates cellular oxygen balance, and induces the expression of hypoxia genes [40]. Hae-Rim et al [41] have found that the serum HIF-1a level in patients with PF is significantly increased, which can be used as an index to judge the severity of IPF and evaluate the therapeutic effect. The PI3K/AKT/mTOR pathway plays an extremely important biological function in cell growth, survival, proliferation, apoptosis, and autophagy [42]. PI3K can be activated by tyrosine kinases to produce PIP3, which can bind to and activate AKT and then activate mTOR [43]. Peiminine is the active ingredient of Fritillariae Thunbergii Bulbus, which can regulate the activity of PI3K/Akt/mTOR pathway to slow down the process of epithelial-mesenchymal transition (EMT) and then inhibit the invasion and migration ability of A549 cells [44]. *β*-Sitosterol-D-glucoside can inhibit the growth and lung metastasis of animal tumors to some extent, upregulate miR-10a expression, inhibit PI3K-AKT signaling pathway, reverse the expression levels of EMT, and inhibit the expression of metastatic related cytokines, eventually inducing apoptosis and inhibit metastasis in tumor cells [45]. Combined with the previous studies [4], YFSJF was found to reduce the expression of CoL-I and CoL-III, reduce the deposition of extracellular matrix, inhibit the phosphorylation of PI3K, Akt, and mTOR, alleviate the pulmonary fibrosis caused by inflammation, activate autophagy, and promote the metabolism and absorption of extracellular matrix such as collagen fiber.

**Figure 11:**
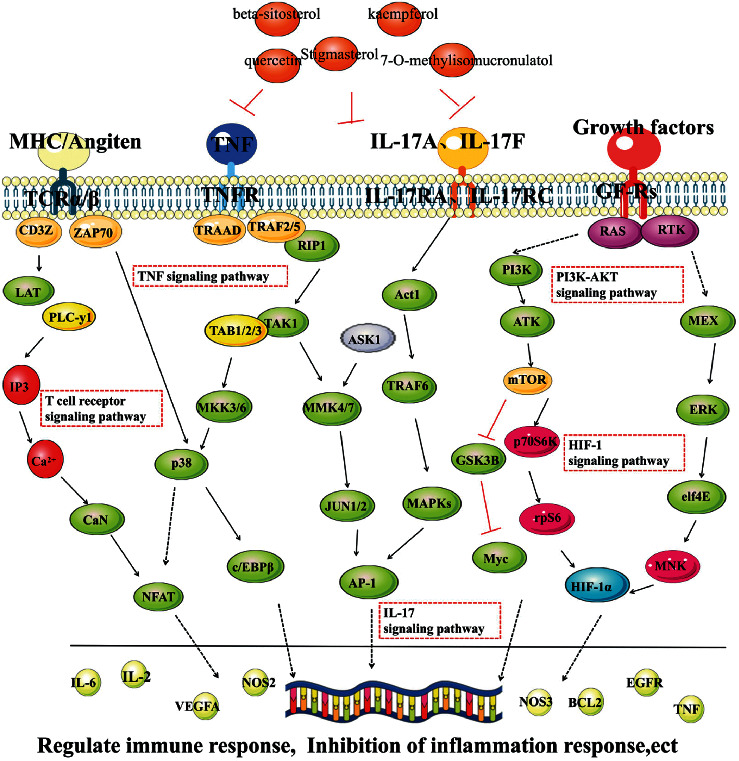
Illustration of the mechanisms of YFSJF in treating PF.

This study shows that quercetin, kaempferol, beta-sitosterol, 7-o-methylosmucronulatol, and stigmasterol can regulate GSK3B, L2, IL6, MAPKs, NOS2, and VEGFA and participate in regulating PI3K-Akt signaling pathway, HIF-1 signaling pathway, and IL-17 signaling pathway. This may be the main material basis for YFSJF to alleviate inflammatory reaction, regulate immunity, inhibit fibroblast proliferation and promote apoptosis, reduce collagen deposition, and finally to alleviate PF. NOS2 and GSK3B are involved in multiple signal transduction pathways and have high degree of freedom in the network of component-target-pathway. Therefore, it can be speculated that YFSJF might regulate signal transduction through NOS2 and GSK3B, thus interfering with PF (Figure 11 and Table 3).

## 5. Conclusions

YFSJF has a therapeutic effect on PF, and the antifibrosis mechanism mainly includes the effects on immunity, inflammatory factors and multiple signaling pathways. This study indicated that quercetin, kaempferol, and beta-sitosterol in the active ingredients of YFSJF could regulate PI3K/AKT signaling pathway, HIF-1 signaling pathway, TNF signaling pathway, IL-17 signaling pathway, T cell receptor signaling pathway, calcium signaling pathway, and Th17 cell differentiation, then alleviate inflammatory response, enhance immunity, inhibit fibroblast proliferation and promote apoptosis, reduce collagen deposition, and eventually alleviate pulmonary fibrosis. The study further revealed the relevant mechanism of YFSJF in preventing and treating PF broadened the clinical application scope of YFSJF and also provided a new therapeutic idea for preventing and treating PF. Due to the limitations of various platform database data and corresponding analysis algorithms and software functions, the results may be biased. In the next step, we will carry out experimental verification based on the results of this study.

## Figures and Tables

**Table 1:** Top 10 basic information of ingredients in YFSJF by their degree values.

MOL Id	Molecule name	Degree	OB	DL	2D structure^*∗*^	Chinese medicine
MOL000098	Quercetin	88	46.43	0.27	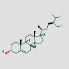	*Hedysarum Multijugum Maxim*
MOL000422	Kaempferol	56	41.88	0.24	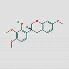	*Hedysarum Multijugum Maxim*
MOL000358	Beta-sitosterol	52	36.91	0.75	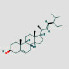	*Fritillariae Thunbrgii Bulbus*
MOL000378	7-O-Methylisomucronulatol	48	74.69	0.29	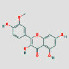	*Hedysarum Multijugum Maxim*
MOL000449	Stigmasterol	45	43.83	0.75	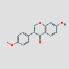	*Panax Notoginseng (Burk.) F. H. Chen Ex C. Chow*
MOL000354	Isorhamnetin	37	49.60	0.30	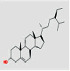	*Hedysarum Multijugum Maxim*
MOL000392	Formononetin	37	69.67	0.21	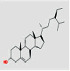	*Hedysarum Multijugum Maxim*
MOL000371	3,9-Di-O-methylnissolin	36	53.71	0.48	None	*Hedysarum Multijugum Maxim*
MOL000296	Hederagenin	35	36.91	0.75	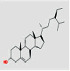	*Curcumae Rhizoma*
MOL000380	(6aR, 11aR)-9, 10-Dimethoxy-6a, 11a-dihydro-6H-benzofurano [3, 2-c] chromen-3-ol	35	64.25	0.42	None	*Hedysarum Multijugum Maxim*

^*∗*^Cited from the DrugBank database.

**Table 2:** Functions of potential target genes based on KEGG analysis.

Id	Term	*P* value	*Q* value	Gene
hsa04933	Age-rage signaling pathway in diabetic complications	9.74*E* − 16	8.41*E* − 14	MMP2/BCL2/CCL2/COL3A1/SELE/IL6/MAPK1/MAPK14/MAPK8/NOS3/PIM1/THBD/F3/JUN/TNF/VCAM1/VEGFA
hsa05418	Fluid shear stress and atherosclerosis	1.86*E* − 14	8.03*E* − 13	MMP2/BCL2/CCL2/TP53/SELE/GSTM1/HMOX1/IFNG/MAPK14/MAPK8/NQO1/NOS3/THBD/PLAT/JUN/TNF/VCAM1/VEGFA
hsa05215	Prostate cancer	1.01*E* − 09	2.90*E* − 08	AR/BCL2/CDK2/TP53/EGFR/GSK3B/IGF1R/MAPK1/EGF/MMP3/PLAT/PLAU
hsa04020	Calcium signaling pathway	5.20*E* − 09	1.12*E* − 07	HTR2A/ADRA1A/ADRA1B/ADRB2/DRD1/EGFR/PRKACA/CHRM1/CHRM2/CHRM3/CHRNA7/NOS2/NOS3/PTGER3/PPP3CA
hsa04657	IL-17 signaling pathway	9.60*E* − 09	1.66*E* − 07	CCL2/GSK3B/IFNG/IL6/MMP1/MAPK1/MAPK14/MAPK8/MMP3/JUN/TNF
hsa01522	Endocrine resistance	1.50*E* − 08	2.16*E* − 07	MMP2/BCL2/TP53/EGFR/ESR2/IGF1R/MAPK1/MAPK14/MAPK8/PRKACA/JUN
hsa04926	Relaxin signaling pathway	2.73*E* − 08	3.37*E* − 07	MMP2/COL3A1/EGFR/MMP1/MAPK1/MAPK14/MAPK8/PRKACA/NOS2/NOS3/JUN/VEGFA
hsa04659	Th17 cell differentiation	3.81*E* − 08	4.11*E* − 07	AHR/IFNG/IL2/IL6/MAPK1/MAPK14/MAPK8/RXRA/RXRB/PPP3CA/JUN
hsa04066	HIF-1 signaling pathway	4.62*E* − 08	4.43*E* − 07	BCL2/EGFR/HMOX1/IGF1R/IFNG/IL6/MAPK1/NOS2/NOS3/EGF/VEGFA
hsa04080	Neuroactive ligand-receptor interaction	5.74*E* − 08	4.96*E* − 07	HTR2A/ADRA1A/ADRA1B/ADRA2A/ADRA2C/ADRB2/OPRD1/DRD1/GABRA1/NR3C1/CHRM1/CHRM2/CHRM3/OPRM1/CHRNA7/ADCYAP1/PTG*E*R3/F2
hsa04151	PI3K-Akt signaling pathway	1.06*E* − 07	8.33*E* − 07	BCL2/CDK2/TP53/EGFR/GSK3B/MET/IGF1R/IL2/IL6/MAPK1/CHRM1/CHRM2/NOS3/PIK3CG/EGF/RBL2/RXRA/VEGFA
hsa05142	Chagas disease (American trypanosomiasis)	2.59*E* − 07	1.86*E* − 06	CCL2/IFNG/IL2/IL6/MAPK1/MAPK14/MAPK8/NOS2/JUN/TNF
hsa01521	EGFR tyrosine kinase inhibitor resistance	2.92*E* − 07	1.94*E* − 06	BCL2/EGFR/GSK3B/MET/IGF1R/IL6/MAPK1/EGF/VEGFA
hsa05219	Bladder cancer	3.88*E* − 07	2.39*E* − 06	MMP2/TP53/EGFR/MMP1/MAPK1/EGF/VEGFA
hsa04022	cGMP-PKG signaling pathway	4.76*E* − 07	2.74*E* − 06	ADRA1A/ADRA1B/ADRA2A/ADRA2C/ADRB2/KCNMA1/PDE3A/OPRD1/MAPK1/NOS3/PIK3CG/PPP3CA
hsa04915	Estrogen signaling pathway	5.25*E* − 07	2.83*E* − 06	MMP2/BCL2/CTSD/EGFR/ESR2/MAPK1/PRKACA/OPRM1/NOS3/PGR/JUN
hsa04668	TNF signaling pathway	6.25*E* − 07	3.18*E* − 06	CCL2/SELE/IL6/MAPK1/MAPK14/MAPK8/MMP3/JUN/TNF/VCAM1
hsa05202	Transcriptional misregulation in cancer	1.51*E* − 06	7.20*E* − 06	TP53/CCNA2/MET/IGF1R/IL6/MPO/PPARG/RXRA/RXRB/MMP3/PLAT/PLAU
hsa05144	Malaria	1.58*E* − 06	7.20*E* − 06	CCL2/SELE/MET/IFNG/IL6/TNF/VCAM1
hsa04660	T cell receptor signaling pathway	3.06*E* − 06	1.32*E* − 05	GSK3B/IFNG/IL2/MAPK1/MAPK14/MAPK8/PPP3CA/JUN/TNF

**Table 3:** Summary of the possible mechanism of YFSJF in intervention of PF.

Chinese medicine	Ingredients	Gene	Pathway
*Hedysarum multijugum maxim/Panax notoginseng (burk.) F. H. Chen ex C. Chowi/Mulberry bark*	Quercetin	ADRB2/EGFR/PRKACA/NOS2/NOS3/PTGER3	Calcium signaling pathway
		CCL2/GSK3B/IFNG/IL6/MMP1/MAPK1/MAPK14/MMP3/JUN/TNF	IL-17 signaling pathway
		AHR/IFNG/IL2/IL6/MAPK1/MAPK14/RXRA/JUN	Th17 cell differentiation
		BCL2/EGFR/HMOX1/IGF1R/IFNG/IL6/MAPK1/NOS2/NOS3/EGF/VEGFA	HIF-1 signaling pathway
		BCL2/CDK2/TP53/EGFR/GSK3B/IGF1R/IL2/IL6/MAPK1/NOS3/PIK3CG/EGF/RBL2/RXRA/VEGFA	PI3K-Akt signaling pathway
		CCL2/SELE/IL6/MAPK1/MAPK14/MMP3/JUN/TNF/VCAM1	TNF signaling pathway
		GSK3B/IFNG/IL2/MAPK1/MAPK14/JUN/TNF	T cell receptor signaling pathway

*Hedysarum Multijugum Maxim*	Kaempferol	ADRA1B/PRKACA/CHRM1/CHRM2/NOS2/NOS3/PPP3CA	Calcium signaling pathway
		GSK3B/MMP1/MAPK14/MAPK8/JUN/TNF	IL-17 signaling pathway
		MAPK14/MAPK8/PPP3CA/JUN	Th17 cell differentiation
		BCL2/HMOX1/IGF1R/NOS2/NOS3	HIF-1 signaling pathway
		BCL2/CDK2/GSK3B/IGF1R/CHRM1/CHRM2/NOS3/PIK3CG	PI3K-Akt signaling pathway
		SELE/MAPK14/MAPK8/JUN/TNF/VCAM1	TNF signaling pathway
		MAPK14/MAPK8/PPP3CA/JUN/TNF	T cell receptor signaling pathway

*Fritillariae Thunbrgii Bulbus*/*Panax Notoginseng (Burk.) F. H. Chen Ex C. Chowi*/*Mulberry bark*	Beta-sitosterol	HTR2A/ADRA1A/ADRA1B/ADRB2/DRD1/PRKACA/CHRM1/CHRM2/CHRM3/CHRNA7/NOS2	Calcium signaling pathway
		GSK3B/MAPK14/JUN	IL-17 signaling pathway
		MAPK14/JUN	Th17 cell differentiation
		BCL2/NOS2	HIF-1 signaling pathway
		BCL2/CDK2/GSK3B/CHRM1/CHRM2/PIK3CG	PI3K-Akt signaling pathway
		MAPK14/JUN	TNF signaling pathway
		GSK3B/MAPK14/JUN	T cell receptor signaling pathway

*Hedysarum Multijugum Maxim*	7-O-Methylisomucronulatol	HTR2A/ADRA1A/ADRA1B/ADRB2/DRD1/PRKACA/CHRM1/CHRM2/CHRM3/NOS2/NOS3	Calcium signaling pathway
		GSK3B/MAPK14	IL-17 signaling pathway
		MAPK14/RXRA/RXRB	Th17 cell differentiation
		NOS2/NOS3	HIF-1 signaling pathway
		CDK2/GSK3B/CHRM1/CHRM2/NOS3/RXRA	PI3K-Akt signaling pathway
		MAPK14	TNF signaling pathway
		GSK3B/MAPK14	T cell receptor signaling pathway

*Panax Notoginseng (Burk.) F. H. Chen Ex C. Chowi*	Stigmasterol	HTR2A/ADRA1A/ADRA1B/ADRB2/PRKACA/CHRM1/CHRM2/CHRM3/CHRNA7/NOS2/NOS3	Calcium signaling pathway
		RXRA	Th17 cell differentiation
		NOS2/NOS3	HIF-1 signaling pathway
		CDK2/CHRM1/CHRM2/NOS3/RXRA	PI3K-Akt signaling pathway

## Supplementary Materials

Supplementary Materials Supplementary Table 1: basic information of ingredients in YFSJF. Supplementary Table 2: the top 20 GO functional categories. Supplementary Table 3: functions of potential target genes based on KEGG analysis.
