# Supplementary material for: A Network Pharmacology Approach to Explore the Potential Mechanisms of Yifei Sanjie Formula in Treating Pulmonary Fibrosis
Source: Evid Based Complement Alternat Med. 2020 Nov 30;2020:8887017. doi: 10.1155/2020/8887017 (PMC7722457; doi:10.1155/2020/8887017)
Supplement: Supplementary Materials — Supplementary Table 1: basic information of ingredients in YFSJF. Supplementary Table 2: the top 20 GO functional categories. Supplementary Table 3: functions of potential target genes based on KEGG analysis. [file 8887017.f1.zip › 8887017.f1/Supplementary Table 1 (1).docx]

Supplementary Table 1 Basic information of ingredients in YFSJF

| ID | Name | OB | DL | Code |
| --- | --- | --- | --- | --- |
| MOL000296 | Hederagenin | 36.91 | 0.75 | CR1,HMM5 |
| MOL000906 | wenjine | 47.93 | 0.27 | CR2 |
| MOL000940 | bisdemethoxycurcumin | 77.38 | 0.26 | CR3 |
| MOL000358 | beta-sitosterol | 36.91 | 0.75 | FTB1,PN2,MC3 |
| MOL001004 | pelargonidin | 37.99 | 0.21 | FTB2 |
| MOL004440 | Peimisine | 57.40 | 0.81 | FTB3 |
| MOL004443 | Zhebeiresinol | 58.72 | 0.19 | FTB4 |
| MOL004444 | Ziebeimine | 64.25 | 0.70 | FTB5 |
| MOL004446 | 6-Methoxyl-2-acetyl-3-methyl-1,4-naphthoquinone-8-O-beta-D-glucopyranoside | 33.31 | 0.57 | FTB6 |
| MOL004450 | Chaksine | 65.63 | 0.66 | FTB7 |
| MOL000033 | (3S,8S,9S,10R,13R,14S,17R)-10,13-dimethyl-17-[(2R,5S)-5-propan-2-yloctan-2-yl]-2,3,4,7,8,9,11,12,14,15,16,17-dodecahydro-1H-cyclopenta[a]phenanthren-3-ol | 36.23 | 0.78 | HMM1,AMK5, |
| MOL000098 | quercetin | 46.43 | 0.28 | HMM2,PN1,MC1 |
| MOL000211 | Mairin | 55.38 | 0.78 | HMM3,MC2 |
| MOL000239 | Jaranol | 50.83 | 0.29 | HMM4 |
| MOL000354 | isorhamnetin | 49.60 | 0.31 | HMM6 |
| MOL000371 | 3,9-di-O-methylnissolin | 53.74 | 0.48 | HMM7 |
| MOL000374 | 5'-hydroxyiso-muronulatol-2',5'-di-O-glucoside | 41.72 | 0.69 | HMM8 |
| MOL000378 | 7-O-methylisomucronulatol | 74.69 | 0.30 | HMM9 |
| MOL000379 | 9,10-dimethoxypterocarpan-3-O-β-D-glucoside | 36.74 | 0.92 | HMM10 |
| MOL000380 | (6aR,11aR)-9,10-dimethoxy-6a,11a-dihydro-6H-benzofurano[3,2-c]chromen-3-ol | 64.26 | 0.42 | HMM11 |
| MOL000387 | Bifendate | 31.10 | 0.67 | HMM12 |
| MOL000392 | formononetin | 69.67 | 0.21 | HMM13 |
| MOL000398 | isoflavanone | 109.99 | 0.30 | HMM14 |
| MOL000417 | Calycosin | 47.75 | 0.24 | HMM15 |
| MOL000422 | kaempferol | 41.88 | 0.24 | HMM16 |
| MOL000433 | FA | 68.96 | 0.71 | HMM17 |
| MOL000438 | (3R)-3-(2-hydroxy-3,4-dimethoxyphenyl)chroman-7-ol | 67.67 | 0.26 | HMM18 |
| MOL000439 | isomucronulatol-7,2'-di-O-glucosiole | 49.28 | 0.62 | HMM19 |
| MOL000442 | 1,7-Dihydroxy-3,9-dimethoxy pterocarpene | 39.05 | 0.48 | HMM20 |
| MOL000449 | Stigmasterol | 43.83 | 0.76 | PN3 |
| MOL001494 | Mandenol | 42.00 | 0.19 | PN4 |
| MOL001792 | DFV | 32.76 | 0.18 | PN5 |
| MOL002879 | Diop | 43.59 | 0.39 | PN6 |
| MOL005344 | ginsenoside rh2 | 36.32 | 0.56 | PN7 |
| MOL007475 | ginsenoside f2 | 36.43 | 0.25 | PN8 |
| MOL000020 | 12-senecioyl-2E,8E,10E-atractylentriol | 62.40 | 0.22 | AMK1 |
| MOL000021 | 14-acetyl-12-senecioyl-2E,8E,10E-atractylentriol | 60.31 | 0.31 | AMK2 |
| MOL000022 | 14-acetyl-12-senecioyl-2E,8Z,10E-atractylentriol | 63.37 | 0.30 | AMK3 |
| MOL000028 | α-Amyrin | 39.51 | 0.76 | AMK4 |
| MOL000049 | 3β-acetoxyatractylone | 54.07 | 0.22 | AMK6 |
| MOL000072 | 8β-ethoxy atractylenolide Ⅲ | 35.95 | 0.21 | AMK7 |
| MOL000011 | (2R,3R)-3-(4-hydroxy-3-methoxy-phenyl)-5-methoxy-2-methylol-2,3-dihydropyrano[5,6-h][1,4]benzodioxin-9-one | 68.83 | 0.66 | SR1 |
| MOL000359 | sitosterol | 36.91 | 0.75 | SR2 |
| MOL001941 | Ammidin | 34.55 | 0.22 | SR3 |
| MOL002644 | Phellopterin | 40.19 | 0.28 | SR4 |
| MOL011730 | 11-hydroxy-sec-o-beta-d-glucosylhamaudol_qt | 50.24 | 0.27 | SR5 |
| MOL011732 | anomalin | 59.65 | 0.66 | SR6 |
| MOL011737 | divaricatacid | 87.00 | 0.32 | SR7 |
| MOL011740 | divaricatol | 31.65 | 0.38 | SR8 |
| MOL011747 | ledebouriellol | 32.05 | 0.51 | SR9 |
| MOL011749 | phelloptorin | 43.39 | 0.28 | SR10 |
| MOL011753 | 5-O-Methylvisamminol | 37.99 | 0.25 | SR11 |
| MOL001697 | Sinoacutine | 63.39 | 0.53 | SS1 |
| MOL010690 | Uniflex BYO | 30.13 | 0.25 | SS2 |
| MOL013037 | 2-(2-phenylethyl)-6-[[(5S,6R,7R,8S)-5,6,7-trihydroxy-4-keto-2-(2-phenylethyl)-5,6,7,8-tetrahydrochromen-8-yl]oxy]chromone | 31.31 | 0.61 | SS3 |
| MOL001474 | sanguinarine | 37.81 | 0.86 | MC4 |
| MOL003758 | Iristectorigenin (9CI) | 71.55 | 0.34 | MC5 |
| MOL003856 | Moracin B | 55.85 | 0.23 | MC6 |
| MOL003857 | Moracin C | 82.13 | 0.29 | MC7 |
| MOL003858 | Moracin D | 60.93 | 0.38 | MC8 |
| MOL003860 | Moracin F | 53.81 | 0.23 | MC9 |
| MOL004912 | Glabrone | 52.51 | 0.50 | MC10 |
| MOL012681 | Dimethyl (methylenedi-4,1-phenylene)biscarbamate | 50.84 | 0.26 | MC11 |
| MOL012686 | 7-methoxy-5,4'-dihydroxyflavanonol | 51.72 | 0.26 | MC12 |
| MOL012689 | cyclomulberrochromene | 36.79 | 0.87 | MC13 |
| MOL012692 | kuwanon D | 31.09 | 0.80 | MC14 |
| MOL012714 | Moracin A | 64.39 | 0.23 | MC15 |
| MOL012717 | moracin M-6,3'-di-O-β-D-glucopyranoside | 37.81 | 0.74 | MC16 |
| MOL012719 | moracin O | 62.33 | 0.44 | MC17 |
| MOL012726 | mulberrofuran G | 92.19 | 0.24 | MC18 |
| MOL012735 | mulberroside C_qt | 71.39 | 0.46 | MC19 |
| MOL012743 | resveratrol-3,4'-di-O-β-D-glucopyranoside | 35.08 | 0.76 | MC20 |
| MOL012749 | sanggenone B | 115.44 | 0.30 | MC21 |
| MOL012753 | sanggenone F | 62.42 | 0.54 | MC22 |
| MOL012755 | sanggenone H | 37.50 | 0.53 | MC23 |
| MOL012760 | sanggenone M | 68.29 | 0.85 | MC24 |
