# Supplementary material for: A Network Pharmacology Approach to Explore the Potential Mechanisms of Yifei Sanjie Formula in Treating Pulmonary Fibrosis
Source: Evid Based Complement Alternat Med. 2020 Nov 30;2020:8887017. doi: 10.1155/2020/8887017 (PMC7722457; doi:10.1155/2020/8887017)
Supplement: Supplementary Materials — Supplementary Table 1: basic information of ingredients in YFSJF. Supplementary Table 2: the top 20 GO functional categories. Supplementary Table 3: functions of potential target genes based on KEGG analysis. [file 8887017.f1.zip › 8887017.f1/Supplementary Table 2 (1).docx]

Supplementary Table 2 The top 20 GO functional categories

| Category | Term | Count | PValue | Genes |  |
| --- | --- | --- | --- | --- | --- |
| GOTERM_BP_DIRECT | GO:0008284~positive regulation of cell proliferation | 19 | 1.07E-10 | EGFR, ODC1, AR, IL6, CDK2, ADCYAP1, IGF1R, MAPK1, CHRM1, BCL2, F2, IFNG, VEGFA, ADRA2A, CHRNA7, EGF, DPP4, HTR2A, IL2 |  |
| GOTERM_BP_DIRECT | GO:0001666~response to hypoxia | 12 | 3.51E-09 | PLAT, VCAM1, KCNMA1, CCL2, HMOX1, VEGFA, CHRNA7, F7, NOS2, MMP2, PLAU, DPP4 |  |
| GOTERM_BP_DIRECT | GO:0045909~positive regulation of vasodilation | 7 | 1.20E-08 | EGFR, PPARD, HMOX1, GJA1, NOS3, NOS2, ADCYAP1 |  |
| GOTERM_BP_DIRECT | GO:0030335~positive regulation of cell migration | 11 | 8.82E-08 | EGFR, MAPK1, IGF1R, DRD1, F10, F3, VEGFA, ADRA2A, F7, HSPA5, PLAU |  |
| GOTERM_BP_DIRECT | GO:0043066~negative regulation of apoptotic process | 15 | 2.60E-07 | EGFR, PPARD, IL6, TP53, PIM1, PDE3A, IGF1R, BCL2, GSK3B, VEGFA, MPO, MAPK8, HSPA5, NQO1, IL2 |  |
| GOTERM_BP_DIRECT | GO:0007568~aging | 10 | 3.91E-07 | VCAM1, IL6, CCL2, SLC6A3, JUN, COL3A1, ADRA1A, MPO, NQO1, HTR2A |  |
| GOTERM_BP_DIRECT | GO:0045944~positive regulation of transcription from RNA polymerase II promoter | 21 | 4.61E-07 | EGFR, AR, IL6, TNF, RXRB, RXRA, MET, PPARG, TP53, NR3C1, AHR, ADCYAP1, PGR, ADRB2, JUN, GSK3B, MAPK14, IFNG, VEGFA, PPP3CA, IL2 |  |
| GOTERM_BP_DIRECT | GO:0070374~positive regulation of ERK1 and ERK2 cascade | 10 | 6.43E-07 | OPRM1, EGFR, IL6, TNF, CCL2, JUN, VEGFA, ADRA1A, HTR2A, ADCYAP1 |  |
| GOTERM_BP_DIRECT | GO:0045907~positive regulation of vasoconstriction | 6 | 9.30E-07 | EGFR, ADRA1B, ADRA1A, GJA1, ADRA2C, HTR2A |  |
| GOTERM_BP_DIRECT | GO:0043406~positive regulation of MAP kinase activity | 7 | 9.89E-07 | PIK3CG, EGFR, TNF, VEGFA, ADRA2A, EGF, HTR2A |  |
| GOTERM_BP_DIRECT | GO:0042493~response to drug | 12 | 1.16E-06 | TOP1, DRD1, IL6, SLC6A3, JUN, BCL2, MAOB, PPARG, IFNG, ADRA1A, PDE3A, HTR2A |  |
| GOTERM_BP_DIRECT | GO:0007165~signal transduction | 22 | 1.58E-06 | EGFR, AR, CCL2, MET, PPARG, NR3C2, GJA1, NR3C1, ESR2, PGR, IGF1R, MAPK1, THBD, CHRM3, CHRM1, MAPK14, ADRA2A, ADRA1A, CHRNA7, EGF, HTR3A, PLAU |  |
| GOTERM_BP_DIRECT | GO:0007271~synaptic transmission, cholinergic | 6 | 1.97E-06 | CHRM3, CHRM2, CHRM1, CHRNA7, HTR3A, NQO1 |  |
| GOTERM_BP_DIRECT | GO:0045471~response to ethanol | 8 | 2.15E-06 | VCAM1, CCL2, SLC6A3, MAOB, HTR3A, NQO1, IL2, ADCYAP1 |  |
| GOTERM_BP_DIRECT | GO:0018107~peptidyl-threonine phosphorylation | 6 | 2.26E-06 | MAPK1, GSK3B, BCL2, PRKACA, CHEK1, MAPK8 |  |
| GOTERM_BP_DIRECT | GO:0006367~transcription initiation from RNA polymerase II promoter | 9 | 2.34E-06 | PGR, AR, PPARD, RXRB, RXRA, PPARG, NR3C2, NR3C1, ESR2 |  |
| GOTERM_BP_DIRECT | GO:0071880~adenylate cyclase-activating adrenergic receptor signaling pathway | 5 | 3.49E-06 | ADRB2, ADRA2A, ADRA1B, ADRA1A, ADRA2C |  |
| GOTERM_BP_DIRECT | GO:0071222~cellular response to lipopolysaccharide | 8 | 3.52E-06 | PPARD, IL6, TNF, CCL2, MAPK14, IFNG, MAPK8, NOS2 |  |
| GOTERM_BP_DIRECT | GO:0051897~positive regulation of protein kinase B signaling | 7 | 7.97E-06 | PIK3CG, EGFR, IL6, TNF, F10, F3, F7 |  |
| GOTERM_BP_DIRECT | GO:0007204~positive regulation of cytosolic calcium ion concentration | 8 | 1.09E-05 | PIK3CG, OPRM1, PTGER3, ADRA1B, ADRA1A, GJA1, IL2, ADCYAP1 |  |
| GOTERM_CC_DIRECT | GO:0005615~extracellular space | 30 | 2.11E-11 | XDH, ACHE, CCL2, TNF, COL3A1, CHEK1, MMP3, MMP2, ADCYAP1, VCAM1, HMOX1, IFNG, EGF, EGFR, PLAT, IL6, LYZ, F7, THBD, F3, VEGFA, F2, PON1, CTSD, MPO, ALOX5, CA2, SELE, PLAU, IL2 |  |
| GOTERM_CC_DIRECT | GO:0005886~plasma membrane | 47 | 2.81E-08 | OPRM1, DRD1, ACHE, TNF, SLC6A3, GJA1, MMP2, VCAM1, IGF1R, HMOX1, ADRA2A, CHRNA7, ADRA2C, PRKACA, NOS3, HSPA5, EGF, HTR3A, SCN5A, DPP4, KCNMA1, EGFR, PIK3CG, AR, F10, GABRA1, PTGER3, MET, PIM1, F7, ADRB2, THBD, CHRM3, CHRM2, F3, CHRM1, GSK3B, F2, ADRA1B, ADRA1A, LTA4H, CA2, KCNH2, SELE, PLAU, OPRD1, HTR2A |  |
| GOTERM_CC_DIRECT | GO:0009986~cell surface | 15 | 8.02E-07 | PLAT, EGFR, ACHE, TNF, SLC6A3, MET, VCAM1, THBD, F3, VEGFA, HSPA5, KCNH2, SCN5A, DPP4, PLAU |  |
| GOTERM_CC_DIRECT | GO:0005901~caveola | 7 | 1.10E-06 | KCNMA1, MAPK1, HMOX1, NOS3, SELE, SCN5A, HTR2A |  |
| GOTERM_CC_DIRECT | GO:0043679~axon terminus | 6 | 4.96E-06 | CCL2, CHRM3, CHRM2, CHRM1, ADRA2C, OPRD1 |  |
| GOTERM_CC_DIRECT | GO:0045121~membrane raft | 9 | 1.22E-05 | OPRM1, EGFR, TNF, SLC6A3, GJA1, CTSD, SELE, DPP4, OPRD1 |  |
| GOTERM_CC_DIRECT | GO:0005576~extracellular region | 23 | 2.12E-05 | PLAT, IL6, ACHE, TNF, F10, CCL2, COL3A1, MET, LYZ, F7, ESR2, MMP3, MMP2, MMP1, ADCYAP1, F2, IFNG, VEGFA, PON1, CTSD, EGF, PLAU, IL2 |  |
| GOTERM_CC_DIRECT | GO:0043235~receptor complex | 7 | 5.33E-05 | EGFR, IGF1R, ADRB2, RXRA, NR3C2, ADRA2A, EGF |  |
| GOTERM_CC_DIRECT | GO:0005887~integral component of plasma membrane | 20 | 1.09E-04 | OPRM1, DRD1, TNF, GABRA1, PTGER3, SLC6A3, MET, GJA1, IGF1R, ADRB2, THBD, CHRM3, CHRM2, CHRM1, ADRA2A, ADRA1B, ADRA1A, ADRA2C, HTR2A, OPRD1 |  |
| GOTERM_CC_DIRECT | GO:0045202~synapse | 7 | 3.69E-04 | EGFR, ACHE, CCL2, GABRA1, CHRM3, CHRM2, CHRM1 |  |
| GOTERM_CC_DIRECT | GO:0032279~asymmetric synapse | 3 | 7.38E-04 | CHRM3, CHRM2, CHRM1 | |
| GOTERM_CC_DIRECT | GO:0045211~postsynaptic membrane | 7 | 8.28E-04 | GABRA1, CHRM3, CHRM2, CHRM1, CHRNA7, HTR3A, OPRD1 | |
| GOTERM_CC_DIRECT | GO:0043204~perikaryon | 5 | 0.002294231 | OPRM1, MAPK1, TOP1, CCL2, IFNG | |
| GOTERM_CC_DIRECT | GO:0031012~extracellular matrix | 7 | 0.004533098 | PLAT, F3, COL3A1, CTSD, HSPA5, MMP2, MMP1 | |
| GOTERM_CC_DIRECT | GO:0005829~cytosol | 29 | 0.004861998 | XDH, PPARG, ADH1C, ADH1B, GJA1, CHEK1, GSTM1, HMOX1, BCL2, PRKACA, NOS3, NOS2, PPP3CA, NQO1, PIK3CG, ODC1, AR, TP53, PDE3A, CDK2, MAPK1, GSK3B, MAPK14, JUN, MAPK8, LTA4H, ALOX5, CA2, HTR2A | |
| GOTERM_CC_DIRECT | GO:0048471~perinuclear region of cytoplasm | 10 | 0.005085285 | EGFR, ODC1, ACHE, CCL2, HMOX1, GSK3B, PPARG, NOS2, KCNH2, SELE | |
| GOTERM_CC_DIRECT | GO:0098794~postsynapse | 3 | 0.005803703 | OPRM1, GSK3B, PPP3CA | |
| GOTERM_CC_DIRECT | GO:0005739~mitochondrion | 15 | 0.008917874 | MAOB, TP53, GJA1, ESR2, MMP2, MAPK1, GSK3B, GSTK1, MAPK14, BCL2, MPO, MAPK8, PRKACA, HSPA5, PPP3CA | |
| GOTERM_CC_DIRECT | GO:0042383~sarcolemma | 4 | 0.009958051 | OPRM1, VCAM1, PPP3CA, SCN5A | |
| GOTERM_CC_DIRECT | GO:1905286~serine-type peptidase complex | 2 | 0.010398922 | F3, F7 | |
| GOTERM_MF_DIRECT | GO:0019899~enzyme binding | 17 | 4.95E-11 | CYP3A4, EGFR, AR, RXRA, PPARG, TP53, CYP1A2, ESR2, PGR, GSTM1, JUN, HMOX1, MAPK14, MAPK8, HSPA5, PPP3CA, SCN5A | |
| GOTERM_MF_DIRECT | GO:0003707~steroid hormone receptor activity | 9 | 8.48E-10 | PGR, AR, PPARD, RXRB, RXRA, PPARG, NR3C2, NR3C1, ESR2 | |
| GOTERM_MF_DIRECT | GO:0008144~drug binding | 9 | 1.03E-08 | PPARD, GABRA1, CHRM3, CHRM2, SLC6A3, CHRM1, PPARG, PPP3CA, HTR2A | |
| GOTERM_MF_DIRECT | GO:0004879~RNA polymerase II transcription factor activity, ligand-activated sequence-specific DNA binding | 7 | 4.60E-08 | AR, PPARD, RXRB, RXRA, PPARG, ESR2, AHR | |
| GOTERM_MF_DIRECT | GO:0005496~steroid binding | 6 | 3.71E-07 | PGR, CYP3A4, AR, NR3C2, NR3C1, ESR2 | |
| GOTERM_MF_DIRECT | GO:0004252~serine-type endopeptidase activity | 11 | 1.70E-06 | PLAT, F10, F3, F2, CTSD, F7, MMP3, MMP2, PLAU, DPP4, MMP1 | |
| GOTERM_MF_DIRECT | GO:0042803~protein homodimerization activity | 17 | 2.88E-06 | XDH, ODC1, ACHE, MAOB, GSTM1, ADRB2, JUN, HMOX1, BCL2, VEGFA, ADRA2A, PON1, CHRNA7, ADRA2C, NOS2, KCNH2, DPP4 | |
| GOTERM_MF_DIRECT | GO:0005102~receptor binding | 12 | 4.66E-06 | PLAT, PGR, AR, CCL2, SLC6A3, GSTK1, F2, GJA1, F7, NOS2, DPP4, ADCYAP1 | |
| GOTERM_MF_DIRECT | GO:0046982~protein heterodimerization activity | 13 | 1.14E-05 | EGFR, PPARD, RXRA, TP53, AHR, BCL2, JUN, VEGFA, ADRA2A, ADRA1B, ADRA1A, ADRA2C, PPP3CA | |
| GOTERM_MF_DIRECT | GO:0005515~protein binding | 71 | 1.23E-05 | OPRM1, PPARD, SLC6A3, PPARG, GJA1, MMP3, MMP2, PGR, TOP1, HMOX1, NOS3, PRKACA, NOS2, NQO1, CCNA2, DPP4, KCNMA1, PIK3CG, EGFR, AR, F10, RBL2, RXRB, RXRA, TP53, PIM1, F7, ESR2, CDK2, AHR, MAPK1, ADRB2, THBD, CHRM3, F3, JUN, F2, VEGFA, CTSD, MAPK8, CA2, KCNH2, DRD1, ACHE, TNF, COL3A1, NR3C2, CHEK1, NR3C1, ADCYAP1, IGF1R, BCL2, GSTK1, ADRA2A, ADRA2C, PPP3CA, HSPA5, EGF, HTR3A, SCN5A, PLAT, ODC1, IL6, MET, MAPK14, GSK3B, LTA4H, ALOX5, SELE, PLAU, OPRD1 | |
| GOTERM_MF_DIRECT | GO:0020037~heme binding | 7 | 1.23E-04 | CYP3A4, HMOX1, PTGS1, MPO, NOS3, CYP1A2, NOS2 | |
| GOTERM_MF_DIRECT | GO:0043565~sequence-specific DNA binding | 12 | 1.56E-04 | PGR, AR, PPARD, RXRB, JUN, RXRA, BCL2, PPARG, NR3C2, TP53, NR3C1, ESR2 | |
| GOTERM_MF_DIRECT | GO:0008134~transcription factor binding | 9 | 1.99E-04 | MAPK1, AR, PPARD, JUN, BCL2, PPARG, PIM1, TP53, AHR | |
| GOTERM_MF_DIRECT | GO:0002020~protease binding | 6 | 2.63E-04 | TNF, F3, SLC6A3, BCL2, TP53, DPP4 | |
| GOTERM_MF_DIRECT | GO:0042166~acetylcholine binding | 4 | 4.09E-04 | ACHE, CHRM3, CHRNA7, HTR3A | |
| GOTERM_MF_DIRECT | GO:0019903~protein phosphatase binding | 5 | 4.35E-04 | EGFR, MAPK14, PPARG, MET, TP53 | |
| GOTERM_MF_DIRECT | GO:0051379~epinephrine binding | 3 | 4.63E-04 | ADRB2, ADRA2A, ADRA2C | |
| GOTERM_MF_DIRECT | GO:0016907~G-protein coupled acetylcholine receptor activity | 3 | 6.46E-04 | CHRM3, CHRM2, CHRM1 | |
| GOTERM_MF_DIRECT | GO:0008270~zinc ion binding | 17 | 7.70E-04 | AR, PPARD, RXRB, RXRA, PPARG, NR3C2, TP53, ADH1C, ADH1B, NR3C1, ESR2, MMP3, MMP2, MMP1, PGR, LTA4H, CA2 | |
| GOTERM_MF_DIRECT | GO:0042802~identical protein binding | 13 | 9.93E-04 | EGFR, MAPK1, IGF1R, TNF, JUN, BCL2, VEGFA, PPARG, TP53, LYZ, KCNH2, NQO1, DPP4 | |
