# Supplementary material for: A Network Pharmacology Approach to Explore the Potential Mechanisms of Yifei Sanjie Formula in Treating Pulmonary Fibrosis
Source: Evid Based Complement Alternat Med. 2020 Nov 30;2020:8887017. doi: 10.1155/2020/8887017 (PMC7722457; doi:10.1155/2020/8887017)
Supplement: Supplementary Materials — Supplementary Table 1: basic information of ingredients in YFSJF. Supplementary Table 2: the top 20 GO functional categories. Supplementary Table 3: functions of potential target genes based on KEGG analysis. [file 8887017.f1.zip › 8887017.f1/Supplementary Table 3 (1).docx]

Supplementary Table 3 Functions of potential target genes based on KEGG analysis.

|  | | |  | |  | |  |  | |  |
| --- | --- | --- | --- | --- | --- | --- | --- | --- | --- | --- |
| ID | Term | | gene | | Chinese medicine | | | pvalue | | qvalue |
| hsa04933 | AGE-RAGE signaling pathway in diabetic complications | | MMP2/BCL2/CCL2/COL3A1/SELE/IL6/MAPK1/MAPK14/MAPK8/NOS3/PIM1/THBD/F3/JUN/TNF/VCAM1/VEGFA | | *Hedysarum Multijugum Maxim*/Atractylodes Macrocephala Koidz/*Saposhnikoviae Radix*/*Fritillariae Thunbrgii Bulbus*/*Mori Cortex*/*Curcumae Rhizoma* | | | 9.74E-16 | | 8.41E-14 |
| hsa05418 | Fluid shear stress and atherosclerosis | | MMP2/BCL2/CCL2/TP53/SELE/GSTM1/HMOX1/IFNG/MAPK14/MAPK8/NQO1/NOS3/THBD/PLAT/JUN/TNF/VCAM1/VEGFA | | *Hedysarum Multijugum Maxim*/Atractylodes Macrocephala Koidz//*Saposhnikoviae Radix*/*Fritillariae Thunbrgii Bulbus*/*Mori Cortex*/*Curcumae Rhizoma* | | | 1.86E-14 | | 8.03E-13 |
| hsa05215 | Prostate cancer | | AR/BCL2/CDK2/TP53/EGFR/GSK3B/IGF1R/MAPK1/EGF/MMP3/PLAT/PLAU | | *Hedysarum Multijugum Maxim*/Atractylodes Macrocephala Koidz/Saposhnikoviae Radix/Fritillariae Thunbrgii Bulbus/Mori Cortex/Panax Notoginseng (Burk.) F. H. Chen Ex C. Chow/Curcumae Rhizoma | | | 1.01E-09 | | 2.90E-08 |
| hsa04020 | Calcium signaling pathway | | HTR2A/ADRA1A/ADRA1B/ADRB2/DRD1/EGFR/PRKACA/CHRM1/CHRM2/CHRM3/CHRNA7/NOS2/NOS3/PTGER3/PPP3CA | | Hedysarum Multijugum Maxim/Atractylodes Macrocephala Koidz/Saposhnikoviae Radix/Fritillariae Thunbrgii Bulbus/Sinapis Semen/Mori Cortex/Panax Notoginseng (Burk.) F. H. Chen Ex C. Chow/Curcumae Rhizomaa | | | 5.20E-09 | | 1.12E-07 |
| hsa04657 | IL-17 signaling pathway | | CCL2/GSK3B/IFNG/IL6/MMP1/MAPK1/MAPK14/MAPK8/MMP3/JUN/TNF | | Hedysarum Multijugum Maxim/Saposhnikoviae Radix/Fritillariae Thunbrgii Bulbus/Mori Cortex/Panax Notoginseng (Burk.) F. H. Chen Ex C. Chow | | | 9.60E-09 | | 1.66E-07 |
| hsa01522 | Endocrine resistance | | MMP2/BCL2/TP53/EGFR/ESR2/IGF1R/MAPK1/MAPK14/MAPK8/PRKACA/JUN | | Hedysarum Multijugum Maxim/Atractylodes Macrocephala Koidz/Saposhnikoviae Radix/Fritillariae Thunbrgii Bulbus/Sinapis Semen/Mori Cortex/Panax Notoginseng (Burk.) F. H. Chen Ex C. Chow | | | 1.50E-08 | | 2.16E-07 |
| hsa04926 | Relaxin signaling pathway | | MMP2/COL3A1/EGFR/MMP1/MAPK1/MAPK14/MAPK8/PRKACA/NOS2/NOS3/JUN/VEGFA | | Hedysarum Multijugum Maxim/Atractylodes Macrocephala Koidz/Saposhnikoviae Radix/Fritillariae Thunbrgii BulbusMori Cortex/Panax Notoginseng (Burk.) F. H. Chen Ex C. Chow/Curcumae Rhizomaa | | | 2.73E-08 | | 3.37E-07 |
| hsa04659 | Th17 cell differentiation | | AHR/IFNG/IL2/IL6/MAPK1/MAPK14/MAPK8/RXRA/RXRB/PPP3CA/JUN | | Hedysarum Multijugum Maxim/Atractylodes Macrocephala Koidz/Saposhnikoviae Radix/Fritillariae Thunbrgii BulbusMori Cortex/Panax Notoginseng (Burk.) F. H. Chen Ex C. Chow/Curcumae Rhizomaa | | | 3.81E-08 | | 4.11E-07 |
| hsa04066 | HIF-1 signaling pathway | | BCL2/EGFR/HMOX1/IGF1R/IFNG/IL6/MAPK1/NOS2/NOS3/EGF/VEGFA | | Hedysarum Multijugum Maxim/Atractylodes Macrocephala Koidz/Saposhnikoviae Radix/Fritillariae Thunbrgii BulbusMori Cortex/Panax Notoginseng (Burk.) F. H. Chen Ex C. Chow/Curcumae Rhizomaa | | | 4.62E-08 | | 4.43E-07 |
| hsa04080 | Neuroactive ligand-receptor interaction | | HTR2A/ADRA1A/ADRA1B/ADRA2A/ADRA2C/ADRB2/OPRD1/DRD1/GABRA1/NR3C1/CHRM1/CHRM2/CHRM3/OPRM1/CHRNA7/ADCYAP1/PTGER3/F2 | | Hedysarum Multijugum Maxim/Atractylodes Macrocephala Koidz/Saposhnikoviae Radix/Fritillariae Thunbrgii Bulbus/Sinapis Semen/Mori Cortex/Panax Notoginseng (Burk.) F. H. Chen Ex C. Chow/Curcumae Rhizomaa | | | 5.74E-08 | | 4.96E-07 |
| hsa04151 | PI3K-Akt signaling pathway | | BCL2/CDK2/TP53/EGFR/GSK3B/MET/IGF1R/IL2/IL6/MAPK1/CHRM1/CHRM2/NOS3/PIK3CG/EGF/RBL2/RXRA/VEGFA | | Hedysarum Multijugum Maxim/Atractylodes Macrocephala Koidz/Saposhnikoviae Radix/Fritillariae Thunbrgii Bulbus/Sinapis Semen/Mori Cortex/Panax Notoginseng (Burk.) F. H. Chen Ex C. Chow/Curcumae Rhizomaa | | | 1.06E-07 | | 8.33E-07 |
| hsa05142 | Chagas disease (American trypanosomiasis) | | CCL2/IFNG/IL2/IL6/MAPK1/MAPK14/MAPK8/NOS2/JUN/TNF | | Hedysarum Multijugum Maxim/Atractylodes Macrocephala Koidz/Saposhnikoviae Radix/Fritillariae Thunbrgii Bulbus/Sinapis Semen/Mori Cortex/Panax Notoginseng (Burk.) F. H. Chen Ex C. Chow/Curcumae Rhizomaa | | | 2.59E-07 | | 1.86E-06 |
| hsa01521 | EGFR tyrosine kinase inhibitor resistance | | BCL2/EGFR/GSK3B/MET/IGF1R/IL6/MAPK1/EGF/VEGFA | | Hedysarum Multijugum Maxim/Atractylodes Macrocephala Koidz/Saposhnikoviae Radix/Fritillariae Thunbrgii Bulbus/Mori Cortex/Panax Notoginseng (Burk.) F. H. Chen Ex C. Chow | | | 2.92E-07 | | 1.94E-06 |
| hsa05219 | Bladder cancer | | MMP2/TP53/EGFR/MMP1/MAPK1/EGF/VEGFA | | Hedysarum Multijugum Maxim/Mori Cortex/Panax Notoginseng (Burk.) F. H. Chen Ex C. Chow | | | 3.88E-07 | | 2.39E-06 |
| hsa04022 | cGMP-PKG signaling pathway | | 4.76E-07 | | Hedysarum Multijugum Maxim/Atractylodes Macrocephala Koidz/Saposhnikoviae Radix/Fritillariae Thunbrgii Bulbus/Sinapis Semen/Mori Cortex/Panax Notoginseng (Burk.) F. H. Chen Ex C. Chow/Curcumae Rhizomaa | | | 4.76E-07 | | 2.74E-06 |
| hsa04915 | Estrogen signaling pathway | | 5.25E-07 | | Hedysarum Multijugum Maxim/Atractylodes Macrocephala Koidz/Saposhnikoviae Radix/Fritillariae Thunbrgii Bulbus/Sinapis Semen/Mori Cortex/Panax Notoginseng (Burk.) F. H. Chen Ex C. Chow/Curcumae Rhizomaa | | | 5.25E-07 | | 2.83E-06 |
| hsa04668 | TNF signaling pathway | | 6.25E-07 | | Hedysarum Multijugum Maxim/Saposhnikoviae Radix/Fritillariae Thunbrgii Bulbus/Sinapis Semen/Mori Cortex/Panax Notoginseng (Burk.) F. H. Chen Ex C. Chow | | | 6.25E-07 | | 3.18E-06 |
| hsa05202 | Transcriptional misregulation in cancer | | 1.51E-06 | | Hedysarum Multijugum Maxim/Atractylodes Macrocephala Koidz/Saposhnikoviae Radix/Fritillariae Thunbrgii Bulbus/Mori Cortex/Panax Notoginseng (Burk.) F. H. Chen Ex C. Chow/Curcumae Rhizomaa | | | 1.51E-06 | | 7.20E-06 |
| hsa05144 | Malaria | | 1.58E-06 | | Hedysarum Multijugum Maxim/Mori Cortex/Panax Notoginseng (Burk.) F. H. Chen Ex C. Chow | | | 1.58E-06 | | 7.20E-06 |
| hsa04660 | T cell receptor signaling pathway | | 3.06E-06 | | Hedysarum Multijugum Maxim/Atractylodes Macrocephala Koidz/Saposhnikoviae Radix/Fritillariae Thunbrgii Bulbus/Sinapis Semen/Mori Cortex/Panax Notoginseng (Burk.) F. H. Chen Ex C. Chow | | | 3.06E-06 | | 1.32E-05 |
